# Supplementary material for: “We are invisible to them”—Identifying the most vulnerable groups in humanitarian crises during the COVID-19 pandemic: The case of Rohingyas and the Host communities of Cox’s Bazar
Source: PLOS Glob Public Health. 2023 Jun 8;3(6):e0000451. doi: 10.1371/journal.pgph.0000451 (PMC10249869; doi:10.1371/journal.pgph.0000451)
Supplement: S1 Checklist — (DOCX) [file pgph.0000451.s001.docx]

**Consolidated criteria for reporting qualitative studies (COREQ): 32-item checklist**

| **No** | **Topic** | **Guide Questions/Description** | **Reported on Page No.** |
| --- | --- | --- | --- |
| **Domain 1: Research team and reflexivity** | | | |
| *Personal Characteristics* | | | |
| 1. | Interviewer/facilitator | Six qualitative researchers including the authors (RS, AAP, MRH) conducted interviews and informal discussions | 13 |
| 2. | Credentials | RS (MSS), AAP (BDS, MSS, MPH), MRH (MSS), BA (MSS, MPH) SFR (PhD) | N/A |
| 3. | Occupation | RS (Senior Research Associate), AAP (Assistant Research Coordinator), MRH (Research Coordinator), BA (Assistant Director), SFR (Dean and Professor of Public Health) | N/A |
| 4. | Gender | Two males and three females were the authors, and four males and two females (RS, AAP, MRH, ASMN, AJT, ZMI) were involved in the data collection | 1, 42 |
| 5. | Experience and training | All the researchers have extensive experience and training with conducting in-depth interviews, key-informant interviews, and informal discussions for qualitative research. | 13 |
| *Relationship with participants* | | | |
| 6. | Relationship established | No | N/A |
| 7. | Participant knowledge of the interviewer | No | N/A |
| 8. | Interviewer characteristics | All interviewers are public health researchers. | 13 |
| **Domain 2: Study design** | | | |
| *Theoretical framework* | | | |
| 9. | Methodological orientation and Theory | Qualitative methods and thematic analysis | 12, 14 |
| *Participant selection* | | | |
| 10. | Sampling | Convenience and snowball sampling | 13 |
| 11. | Method of approach | Face-to-face | 13 |
| 12. | Sample size | 24 | 12 |
| 13. | Non-participation | None. All the participants agreed to give an interview after we approached them. | 13 |
| *Setting* | | | |
| 14. | Setting of data collection | Each interview was conducted in a private place (mostly inside their house) selected by the participant | 14 |
| 15. | Presence of non-participants | None of the non-participants was present during the interviews since the researchers politely diverted the curious community bystanders, and sometimes enumerators conducted diversion interviews to keep them busy. | 14 |
| 16. | Description of sample | See the profile of the participants | 15 |
| *Data collection* | | | |
| 17. | Interview guide | The interview guidelines were pre-developed, pretested, and finalized prior conducting the interviews | 13 |
| 18. | Repeat interviews | None | N/A |
| 19. | Audio/visual recording | All the interviews were audio-recorded | 13 |
| 20. | Field notes | Yes | 13 |
| 21. | Duration | The average length of the interviews was 50 minutes | 13 |
| 22. | Data saturation | Yes | 13 |
| 23. | Transcripts returned | None of the transcripts were returned to the participants since most of them were unable to read and write in English, or Bangla | 14 |
| **Domain 3: Analysis and findings** | | | |
| *Data analysis* | | | |
| 24. | Number of data coders | Six | 14 |
| 25. | Description of the coding tree | A coding tree was not developed. | N/A |
| 26. | Derivation of themes | Themes were derived from the data | 14 |
| 27. | Software | Atlas Ti, qualitative software, version 9.0 was used for the analysis | 14 |
| 28. | Participant checking | No | N/A |
| *Reporting* | | | |
| 29. | Quotations presented | Yes | 18-30 |
| 30. | Data and findings consistent | Several relevant quotations used to illustrate findings. | 18-30 |
| 31. | Clarity of major themes | Yes | 15-30 |
| 32. | Clarity of minor themes | Yes | 15-30 |
