# Supplementary material for: “We are invisible to them”—Identifying the most vulnerable groups in humanitarian crises during the COVID-19 pandemic: The case of Rohingyas and the Host communities of Cox’s Bazar
Source: PLOS Glob Public Health. 2023 Jun 8;3(6):e0000451. doi: 10.1371/journal.pgph.0000451 (PMC10249869; doi:10.1371/journal.pgph.0000451)
Supplement: Attachment — Submitted filename: Response to Reviewers.docx [file pgph.0000451.s002.docx]

The Editor and Reviewers,

PLOS Global Public Health

Date: January 8, 2023

**Subject:** Rebuttal Letter for the Manuscript titled “We are invisible to them” - Identifying the most vulnerable groups in humanitarian crises during the COVID-19 pandemic: The Case of Rohingyas and the Host communities of Cox’s Bazar.

Dear Editors & Reviewers,

Thank you for your feedback that has helped us strengthen the manuscript. Here are the redressal and specific comments to your feedback:

|  | **Comment** | **Redressal** |
| --- | --- | --- |
| Journal-related comments | | |
| 1. | Please amend your detailed online Financial Disclosure statement. This is published with the article. It must therefore be completed in full sentences and contain the exact wording you wish to be published.  **a.** State the initials, alongside each funding source, of each author to receive each grant. **b.** State what role the funders took in the study. If the funders had no role in your study, please state: “The funders had no role in study design, data collection and analysis, decision to publish, or preparation of the manuscript. | Thank you for the comment.  The funding was done by BRAC James P Grant School of Public Health, BRAC University   a) BRAC James P Grant School of Public Health, BRAC University funded the research project. b) The funders had no role in study design, data collection and analysis, decision to publish, or preparation of the manuscript |
| 2. | In the Funding Information you indicated that no funding was received. Please revise the Funding Information field to reflect funding received.  Please ensure that the funders and grant numbers match between the Financial Disclosure field and the Funding Information tab in your submission form. Note that the funders must be provided in the same order in both places as well. | The funding information has been updated to match with the Financial Disclosure Statement |
| 3. | Please update your online Competing Interests statement. If you have no competing interests to declare, please state: “The authors have declared that no competing interests exist.” | Thank you for the comment.  Online Competing Interests statement has been updated in lines number 26-27 |
| 4. | Please provide separate figure files in .tif or .eps format and remove any figures embedded in your manuscript file. Please also ensure that all files are under our size limit of 10MB. | No figure was added. We formatted the table according to journal requirements. |
| 5. | For more information about how to convert your figure files please see our guidelines | The table were converted according to the journal format |
| Comments to the Author – Reviewer 1 | | |
| 1. | From what I understand, this paper sets out to identify the most vulnerable groups within both the Rohingya and host communities of cox bazaar. It does so using 3 separate pieces of information. It identifies many groups as vulnerable.  However, what I feel the study fails to do is accomplish its main task – that is to identify THE MOST vulnerable group. Instead, it describes many forms of vulnerability that as a result affect a large swath of the population. COVID-19 is an additional element in the study but its role further complicates rather than focuses the discussion around vulnerability. As such, given the findings are so broad, I don’t know what I learn from the study. | Thank you for the review. The results section has been reorganized, reanalysed, and edited for the better clarity (Line number- 235-531). Also, a table is added in the methodology section which showed how many and what kind of MVGs were identified through three techniques (Line number- 170). By doing so, we could streamline the result section as well |
| 2. | The study makes recommendations but I felt these were premature without a more detailed discussion around what it means to be most vulnerable and thus how one would want to prioritise policy attention. | Thank you for the review.  The result section has been reorganized with focused discussion around the vulnerability, and vulnerable groups, and discussion section has been also updated according to the results (Line number-- 235-531, 532-605). |
| 3. | I think there is excellent data underlying this paper but without more focus, it is too long and does not make a central argument or present really insightful findings. The authors could revise the paper with a more focused approach and make a more meaningful contribution to the literature. | Thank you for the review. The paper has been revised accordingly. |
| Comments to the Author – Reviewer 2 | | |
| 1. | The introduction sets the article up as a comparison between the host communities and the Rohingya refugees, but the analysis itself and resulting discussion is not situated as a comparison between the two populations -- i.e., presenting and discussing why particular subgroups are more vulnerable in one setting than the other, highlighting cases where populations are vulnerable in both settings. Alternatively, focus in on one population alone (e.g., Rohingya). As the paper is currently written, the analysis and discussion does not speak to the introduction.  I also think the introduction needs a paragraph outlining what the study is, how it was implemented, and what it is contributing to the literature | Thank you for the review. The results section has been reorganized, reanalysed and presented with a comparison between the two populations (Lines number-235-531).  A paragraph outlining what the study is, how it was implemented, and what it is contributing to the literature has been added (Lines number- 141-151) |
| 2. | The introduction needs to be significantly streamlined and updated. there seems to be some literature missing in the introduction that could help fram the article and better situate the contribution within the literature. For example:  Barua A, Karia RH. Challenges Faced by Rohingya Refugees in the COVID-19 Pandemic. Annals of Global Health. 2020; 86(1): 129, 1–3. DOI: <https://doi.org/10.5334/aogh.3052>  Gulglielmi, S., Seager, J., Mitu, K., Baird, S., Jones, N. Exploring the impacts of COVID-19 on Rohingya adolescents in Cox's Bazar: A mixed-methods study. Journal of Migration and Health. 2020; 1-2. <https://doi.org/10.1016/j.jmh.2020.100031>  Zakir Hossain, ANM. Sustainable Development and Livelihoods of Rohingya Refugees in Bangladesh: The Effects of COVID-19. International Journal of Sustainable Development and Planning. 2021; 16(6): 1141-1152.  The latter of the three could nicely highlight that policy responses are inadequate and this article contributes by identifying most at risk groups. | Thank you for the review and suggestions of updated literature.  The introduction has been streamlined and updated (Line number-81-151). |
| 3. | A few other places that need citations; a) Sentence at line 89 "Evidence as shown... b)Sentence at line 92 "Moroever, the general perception..."  c) Line 123 "Since the introduction of.." is there not anything more recent than July 2020 at this point? | Thank you for the review   a) Citation has been added in line number- 95 b) Citation has been added in line number- 98 c) We have removed that part as our result section has been significantly streamlined and reorganized with more focus. Hence, this information was not required based on the edit we made in results sections. |
| 4. | In general the paper should be edited for clarity. In several locations there is awkward phrasing, e.g.: a) In the abstract "Covid-19 pandemic has had generally an adverse impact..." I would either drop the generally or move it to be "has had a generally adverse impact"  b) First sentence of introduction. Should have commas around "also referred to...(FDMN). and "who reside" should be "that reside"  c) line 83 "On the other hand' -- there is not really a juxtaposition being presented here so strange to say  d) Line 111 - suggest switching the 'on the other hand' to "at the same time" | Thank you for the review. Required edits have been made for better clarity.   a) Changed accordingly (Line number-34) b) Added the comma in line number- 82 and replaced “Who reside” with “that reside” in line number- 83 c) Removed in line number- 87 d) Switched ‘on the other hand' to "at the same time" in line number- 116 |
| 5. | In the abstract: Sentence starting "The finalized list of the most vulnerable groups..." the ; after methods should be a : l I would also use a : in the results section "identified in this study were: single | Thank you for the review  Changes have been made in line number- 55. |
| 6. | Table 1 is very difficult to read. | Removed this table and added a new one as we considered this one more necessary to understand the identification of the MVGs (Line number- 170) |
| 7. | Suggest having a copy-editor go through the paper. E.g.,:  a) line 120, "escalated from the stigma" - delete the?  b) line 140 "."r instance"  c) line 175 "local communities" Do you mean leaders | Edited accordingly   a) removed that part b) Fixed accordingly (line number- 129-130)   c) "local communities" include community people; youth, adolescents, adult, and also community leaders. Changed with community people (Line number-166) |

Thank you for providing us with a learning opportunity with these comments. We are hopeful that the pre-peer-review feedback has helped us reinforce our manuscript and concise it to comply with Journal’s aims and scope.

We look forward to hearing from you soon.

Thank you,

Best regards,

Corresponding Author

Rafia Sultana
