# Supplementary material for: “We are invisible to them”—Identifying the most vulnerable groups in humanitarian crises during the COVID-19 pandemic: The case of Rohingyas and the Host communities of Cox’s Bazar
Source: PLOS Glob Public Health. 2023 Jun 8;3(6):e0000451. doi: 10.1371/journal.pgph.0000451 (PMC10249869; doi:10.1371/journal.pgph.0000451)
Supplement: Attachment — Submitted filename: Response to Reviewers.docx [file pgph.0000451.s003.docx]

The Editor,

PLOS Global Public Health

Date: March 15, 2023

**Subject:** Rebuttal Letter for the Manuscript titled “We are invisible to them” - Identifying the most vulnerable groups in humanitarian crises during the COVID-19 pandemic: The Case of Rohingyas and the Host communities of Cox’s Bazar.

Dear Editors and reviewers,

Thank you for your feedback that has helped us strengthen the manuscript. Here are the redressal and specific comments to your feedback:

| **Comment** | **Redressal** |
| --- | --- |
| **Section One: Comments to Author** | |
| PLOS Global Public Health considers qualitative and mixed-methods studies for publication. We recommend that authors use the COREQ checklist, or other relevant checklists listed by the Equator Network, such as the SRQR, to ensure complete reporting (<http://journals.plos.org/globalpublichealth/s/submission-guidelines#loc-qualitative-research>).   In general, we would expect qualitative studies to include the following: 1) defined objectives or research questions; 2) description of the sampling strategy, including rationale for the recruitment method, participant inclusion/exclusion criteria and the number of participants recruited; 3) detailed reporting of the data collection procedures; 4) data analysis procedures described in sufficient detail to enable replication; 5) a discussion of potential sources of bias; and 6) a discussion of limitations. | Thank you for the review. Methodology section has been revised according to the COREQ checklist and journal guideline.   1) See defined objective in line number 152-154 2) Description of the sampling strategy, including rationale for the recruitment method, participant inclusion/exclusion criteria and the number of participants recruited were added in line number 207-213, 2018-221 3) See the detailed reporting of the data collection procedures in line number 214-225 4) See data analysis procedures described in sufficient detail to enable replication in line number 226-233   5 & 6) Given the length, we considered not to include this in the methodology. |
| **Section Two: Journal Requirements** | |
| Please review your reference list to ensure that it is complete and correct. If you have cited papers that have been retracted, please include the rationale for doing so in the manuscript text, or remove these references and replace them with relevant current references. Any changes to the reference list should be mentioned in the rebuttal letter that accompanies your revised manuscript. If you need to cite a retracted article, indicate the article’s retracted status in the References list and also include a citation and full reference for the retraction notice. | Reviewed Accordingly. No new reference was added or retracted in this version. |

Thank you for providing us with a learning opportunity with these comments. We are hopeful that your feedback has helped us reinforce our manuscript and concise it to comply with Journal’s aims and scope.

We look forward to hearing from you soon.

Thank you,

Best regards,

Corresponding Author

Rafia Sultana
