# Supplementary material for: “We are invisible to them”—Identifying the most vulnerable groups in humanitarian crises during the COVID-19 pandemic: The case of Rohingyas and the Host communities of Cox’s Bazar
Source: PLOS Glob Public Health. 2023 Jun 8;3(6):e0000451. doi: 10.1371/journal.pgph.0000451 (PMC10249869; doi:10.1371/journal.pgph.0000451)
Supplement: Attachment — Submitted filename: Response to Reviewers.docx [file pgph.0000451.s004.docx]

The Editor,

PLOS Global Public Health

Date: April 25, 2023

**Subject:** Rebuttal Letter for the Manuscript titled “We are invisible to them” - Identifying the most vulnerable groups in humanitarian crises during the COVID-19 pandemic: The Case of Rohingyas and the Host communities of Cox’s Bazar.

Dear Editors and reviewers,

Thank you for your feedback that has helped us strengthen the manuscript. Here are the redressal and specific comments to your feedback:

| **Comment** | **Redressal** |
| --- | --- |
| **Section One: Comments to Author** | |
| A completed COREQ checklist will be a prerequisite for acceptance | Thank you for the review. A completed COREQ checklist is prepared and also submitted accordingly. |
| The manuscript has only been revised according to some of the comments given. The response that some of the comments not are addressed because of the length of the manuscript, are not satisfactory, this is an online journal and page printing cost is not a relevant issue to raise. Therefore, please address: 5) a discussion of potential sources of bias; and 6) a discussion of limitations. | Thank you for your feedback. A discussion of potential sources of bias; and a discussion of limitations were added in line number 646-662 |
| **Section Two: Journal Requirements** | |
| Please review your reference list to ensure that it is complete and correct. If you have cited papers that have been retracted, please include the rationale for doing so in the manuscript text, or remove these references and replace them with relevant current references. Any changes to the reference list should be mentioned in the rebuttal letter that accompanies your revised manuscript. If you need to cite a retracted article, indicate the article’s retracted status in the References list and also include a citation and full reference for the retraction notice | Reviewed Accordingly. No new reference was added or retracted in this version. |

Thank you for providing us with a learning opportunity with these comments. We are hopeful that your feedback has helped us reinforce our manuscript and concise it to comply with Journal’s aims and scope.

We look forward to hearing from you soon.

Thank you,

Best regards,

Corresponding Author

Rafia Sultana
